# Construction of an Isonucleoside on a 2,6-Dioxobicyclo[3.2.0]-heptane Skeleton

**DOI:** 10.3390/molecules20034623

**Published:** 2015-03-12

**Authors:** Yuichi Yoshimura, Satoshi Kobayashi, Hitomi Kaneko, Takeshi Suzuki, Tomozumi Imamichi

**Affiliations:** 1 Faculty of Pharmaceutical Sciences, Tohoku Pharmaceutical University, 4-4-1 Komatsushima, Aoba-ku, Sendai 981-8558, Japan; E-Mails: s-kobayashi@fujichemical.co.jp (S.K.); hitomi.f528@gmail.com (H.K.); suzutake19851109@coral.plala.or.jp (T.S.); 2 Laboratory of Human Retrovirology, Leidos Biochemical Research Inc., Frederick National Laboratory for Cancer Research, Frederick, MD 21702, USA; E-Mail: timamichi@mail.nih.gov

**Keywords:** nucleoside, bicyclo, oxetane ring, conformation

## Abstract

We have built a new isonucleoside derivative on a 2,6-dioxobicyclo[3.2.0]heptane skeleton as a potential anti-HIV agent. To synthesize the target compound, an acetal-protected dihydroxyacetone was first converted to a 2,3-epoxy-tetrahydrofuran derivative. Introduction of an azide group, followed by the formation of an oxetane ring, gave a pseudosugar derivative with a 2,6-dioxobicyclo[3.2.0]heptane skeleton. The desired isonucleoside was obtained by constructing a purine base moiety on the scaffold, followed by amination.

## 1. Introduction

Since the discovery of 3'-azidothymidine (AZT), much attention has been paid to the development of effective chemotherapeutic agents against the human immunodeficiency virus (HIV), a causative agent for AIDS [1,2]. More than 20 anti-HIV drugs have now been approved and are clinically used for the treatment of AIDS. Among them, nucleoside reverse transcriptase inhibitors (NRTIs) play a critical role in the treatment of AIDS patients. In the most successful regimen for AIDS referred to as ART (Anti-Retroviral Therapy), a cocktail of anti-HIV drugs, including NRTIs, non-nucleoside reverse transcriptase inhibitors (NNRTIs), and protease inhibitors (PIs) [3], is used. Although ART greatly contributes to increasing the lifespan of patients, drug-resistant strains of the virus are still a serious problem [4,5]. Therefore, new drugs that are effective against the resistant virus strains are constantly needed.

**Figure 1:**
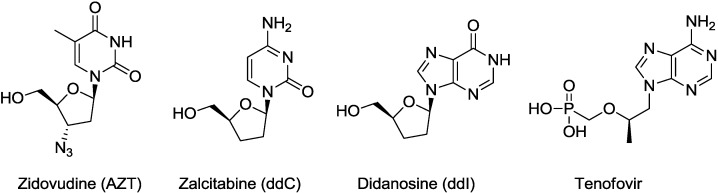
Approved NRTIs.

**Figure 2:**
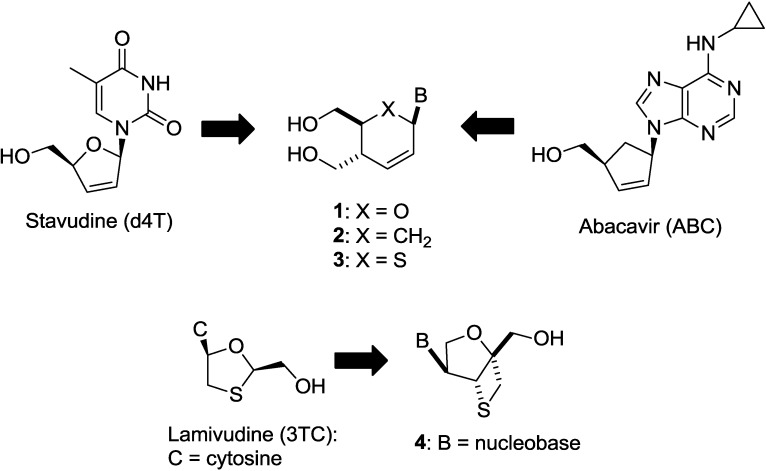
Our previous works searching for anti-HIV nucleosides built on a new scaffold.

Most NRTIs belong to a category of dideoxynucleosides, e.g., zalcitabine (ddC) [6] and didanosine (ddI) [6]. AZT [7] and lamivudine [8] are 3'-substituted dideoxynucleoside derivatives, and abacavir is a carbocyclic analogue of dideoxynucleoside. Only tenofovir [9], which is a nucleoside phosphonate (Figure 1 and Figure 2), is different. From the viewpoint of designing new anti-HIV agents, nucleosides constructed on a novel scaffold are expected to have antiviral activity against the resistant virus strains and may avoid cross-resistance to the known NRTIs.

**Figure 3:**
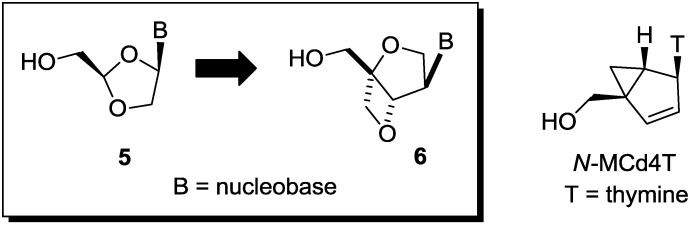
Design of nucleoside derivative built on a 2,6-bicyclo[3.2.0]heptane skeleton.

Thus, we have been focusing on the design and synthesis of nucleoside derivatives attached to a pseudosugar scaffold [10,11,12,13,14,15,16,17]. Among them, nucleosides with cyclohexenyl [13], dihydrothiophenyl [15], and dihydropyranyl [17] groups in place of a furanose ring have been synthesized as “ring-expanded” analogues of stavudine and abacavir. Dihydropyranyl derivative **1** did not show any activity, whereas cyclohexenyl derivative **2** showed weak anti-HIV activity [13,17]. On the other hand, dihydrothiophenyl derivative **3** showed significant anti-HIV activity [15]. In addition, we have applied the “ring-expanding” concept to lamivudine and synthesized isonucleosides **4** constructed on 2-oxa-6-thiabicyclo[3.2.0]heptane [14]. The isonucleoside **4** was also considered as conformationally-restricted analogue of lamivudine by introducing a fused thietane ring (*vide infra*). However, **4** showed no anti-HIV activity (Figure 2). In this study, we planned to build isonucleoside **6** on a 2,6-dioxobicyclo[3.2.0]heptane skeleton, an analogue of dioxolane nucleoside **5** which exhibited potent anti-HIV activity [18,19,20]. The similar conformationally-restricted analogue of d4T was known: cyclopropane-fused carbocyclic d4T (*N*-MCd4T), fixed in north conformation, was originally reported by Marquez and his colleagues and had significant anti-HIV activity with lesser cytotoxicity [21]. In addition, D-enantiomer of **5** was known to have potent cytotoxicity [18,19,20]. Thus, isonucleoside **6** should be promising although the thietane-fused derivative **4** was inactive (Figure 3).

## 2. Results and Discussion

**Scheme 1:**
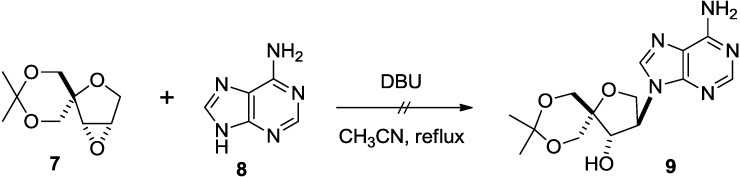
Attempt to introduce adenine moiety.

Following our previous reports [11,14], epoxide **7** was synthesized. We first attempted to introduce an adenine onto **7** by treating it with DBU [22]. However, the reaction did not give the desired product **9** (Scheme 1).

**Scheme 2:**
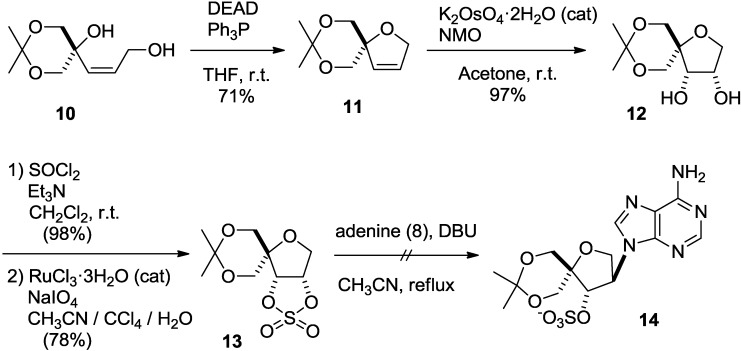
Second attempt to introduce adenine using a cyclic sulfate.

In addition, Lewis acid-catalyzed reactions did not afford **9** either (data not shown). Since the low reactivity of **7** might be due to its rigid structure, we next tried nucleophilic substitution using a more reactive cyclic sulfate derivative [23]. *Cis*-allyl alcohol **10**, a precursor of epoxide **7** [11,14], was cyclized under Mitsunobu conditions, as in the case of epoxide **7** [11,14], to give dihydrofuran **11** in 71% yield. Treatment of dihydrofuran **11** with potassium osmate in the presence of *N*-methylmorpholine *N*-oxide afforded *cis*-diol **12**. The desired cyclic sulfate **13** was obtained by treatment of **12** with thionyl chloride, followed by oxidation. However, the nucleophilic substitution of **13** with adenine did not afford the desired isonucleoside **14** (Scheme 2).

**Scheme 3:**
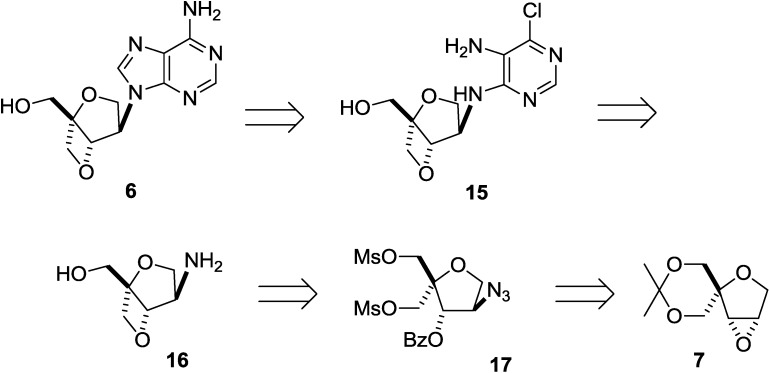
Revised retrosynthesis of isoadenosine **6** built on a 2,6-dioxobicyclo[3.2.0]heptane skeleton.

Therefore, we revised our plan to synthesize an isoadenosine constructed on a 2,6-dioxobicyclo[3.2.0]heptane scaffold, and the revised scheme is shown in Scheme 3 in a retrosynthetic manner. Instead of the direct introduction of adenine, we decided to build the adenine ring on the 2,6-dioxobicyclo[3.2.0]heptane pseudosugar skeleton in a stepwise manner. According to this plan, compound **16** was thought to be a suitable intermediate for preparing **15** since it can be transformed to **6** by the formation of an imidazole ring, followed by amination. Fused oxetane derivative **16** can be obtained from dimesylate **17**. Finally, epoxide **7**, described above, was selected as the starting compound because it can be converted to **17** by the selective cleavage of the oxirane ring with an azide anion (Scheme 2).

**Scheme 4:**
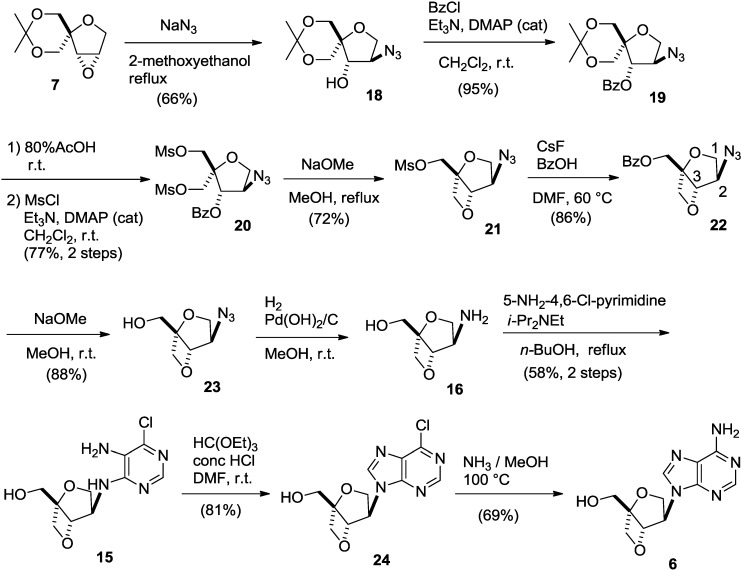
Synthesis of isoadenosine **6**.

First, regioselective cleavage of the oxirane ring of **7** with sodium azide in 2-methoxyethanol under reflux conditions gave the desired azide-alcohol **18** as a single regioisomer in 66% yield. It is obvious that the nucleophilic azide anion attacked from the less hindered side since similar regioselective epoxide opening was observed in our previous report [11,14]. After benzoylation of the hydroxyl group, the acetal group of **19** was removed by using acidic hydrolysis, and the resulting diol was mesylated to give dimesylate **20** in good yield. Deprotection of the benzoyl group and the subsequent formation of an oxetane ring were achieved by treating **20** with sodium methoxide under reflux conditions to give mesylate **21** in 72% yield. The structure of **21** was unambiguously determined by comparison of 1D NMR spectrum with that of 2-oxa-6-thiabicyclo[3.2.0]heptane skeleton [14] after converting it to benzoate **22** by treatment with benzoic acid in the presence of cesium fluoride. In ^1^H-NMR spectra of **22**, the peaks corresponding to the methyl groups of the dimesylate were absent, and only the peaks corresponding to the benzoyl group in the range of 8.1–7.4 ppm were present. In addition, one of the methylene protons at the 2-position was observed as a doublet at 4.42 ppm, meaning that the coupling with H-3 disappeared. This indicates that the conformation around the tetrahydrofuran ring changes and becomes fixed, which causes a loss of coupling between one pair of H-2 and H-3 protons. A similar correlation between conformation and couplings in ^1^H-NMR spectra has been reported for the 2-oxa-6-thiabicyclo[3.2.0]heptane skeleton [14]. Moreover, in the mass spectrum of the compound, a molecular ion peak was observed at *m/z* = 276, further supporting the assignment of the structure. Finally, **22** was deprotected to afford azido-alcohol **23** in 88% yield (Scheme 4).

Azido-alcohol **23** was reduced by catalytic hydrogenation to give key intermediate **16**, which was treated with 5-amino-4,6-dichloropyrimidine and diisopropylethylamine in refluxing *n*-butanol [23] to give diaminopyrimidine derivative **15** in 58% yield from **23**. Formation of the imidazole ring of **15** was accomplished by treatment with orthoethyl formate under acidic conditions [24] to give 6-chloropurine nucleoside **24**. Finally, the isoadenosine was built on the 2,6-dioxobicyclo[3.2.0]heptane scaffold **6** by heating **24** with methanolic ammonia in a sealed tube in 69% yield (Scheme 4). Isoadenosine **6** did not show any significant activity against HIV even at a concentration of 100 µM.

## 3. Experimental Section

### General Information

Melting points are uncorrected. NMR spectra were recorded at 400 MHz (^1^H), 100 MHz (^13^C) using CDCl_3_ as a solvent. As an internal standard, tetramethylsilane was used for CDCl_3_. Mass spectra were obtained by EI or FAB mode. Silica gel for chromatography was Silica Gel 60N (spherical, neutral, 100–210 µm, Kanto Chemical Co. Inc., Tokyo, Japan). When the reagents sensitive to moisture were used, the reaction was performed under argon atmosphere.

*8,8-Dimethyl-1,7,9-trioxaspiro[4,5]dec-3-ene* (**11**). To a solution of PPh_3_ (2.49 g, 9.48 mmol) in THF (10 mL) was added DEAD (4.31 mL, 9.48 mmol) and the mixture was stirred at room temperature for 5 min. To this mixture, a solution of **10** [11,14] (1.04 g, 5.58 mmol) in THF (10 mL) was added. The mixture was stirred at room temperature for 1 h. After the solvent was removed under reduced pressure, the residue was purified by silica gel column chromatography (hexane–ethyl acetate = 19:1) to give **11** (677 mg, 71%) as a white crystal. mp 47–49 °C; ^1^H-NMR (CDCl_3_) δ 1.45 (3H, s), 1.48 (3H, s), 3.76 (2H, d, *J* = 11.6 Hz), 3.81 (2H, d, *J* = 11.6 Hz), 4.71 (2H, t, *J* = 1.9 Hz), 5.84-5.87 (1H, m), 6.02 (1H, d, *J* = 6.3 Hz); ^13^C-NMR (CDCl_3_) δ 22.6, 24.6, 66.7, 74.9, 98.0, 128.1, 129.1; IR (neat) 2924.2, 2853.1, 1724.1, 1215.9, 758.3 cm^−1^; FAB-MS (*m/z*) 155 [M−15]^+^; Anal. Calcd for C_9_H_14_O_3_; C, 62.84; H, 8.32. Found; C, 62.78; H, 8.44.

(3S*,4S*)-8,8-Dimethyl-1,7,9,-trioxaspiro[4.5]decane-3,4-diol (**12**). To a solution of **11** (73 mg, 0.43 mmol) and NMO (0.22 mL) in acetone (4 mL), was added a solution of K_2_OsO_4_·2H_2_O (1 mg, 0.004 mmol) in H_2_O (0.4mL) at 0 °C. After stirred at room temperature for 60 h, Na_2_S_2_O_3_·5H_2_O (125 mg) was added and the mixture was stirred at room temperature for 30 min. After the whole mixture was dried over Na_2_SO_4_, the solid materials were removed by suction and washed with ethyl acetate. The combined filtrate was concentrated under reduced pressure. The residue was purified by silica gel column chromatography (CHCl_3_–MeOH = 19:1) to give **12** (84 mg, 97%). ^1^H-NMR (CDCl_3_) δ 1.43 (3H, s), 1.49 (3H, s), 3.59 (1H, dd, *J* = 11.6, 1.9 Hz), 3.80 (1H, d, *J* = 9.7 Hz), 3.82–3.87 (2H, m), 3.94 (1H, dd, *J* = 9.7, 4.9 Hz), 4.14 (1H, dd, *J* = 11.6, 1.9 Hz), 4.22 (1H, d, *J* = 5.3 Hz) 4.34 (1H, q, *J* = 5.0 Hz); ^13^C-NMR (CDCl_3_) δ 21.0, 25.8, 63.3, 66.5, 71.0, 71.4, 74.9, 76.7, 98.4; IR (KBr) 3306.4, 2953.6, 2741.6, 1452.2, 524.19 cm^−1^; EI-MS (*m/z*): 204 [M+1]^+^; HRMS Calcd for C_9_H_15_N_3_O_4_: 204.0998, Found: 204.0992.

(3S*,4S*)-8,8-Dimethyl-1,7,9,-trioxaspiro[4.5]decane-3,4-cyclicsulfate (**13**). To a solution of **12** (410 mg, 2.01 mmol) and Et_3_N (672 µL, 4.82 mmol) in CH_2_Cl_2_ (10 mL), was added dropwise a solution of SOCl_2_ (113 μL, 1.55 mmoL) in CH_2_Cl_2_ (10 mL) at 0 °C. After stirred at room temperature for 15 min, the mixture was washed with water. The water layer was extracted with CHCl_3_ twice and the combined organic layer was washed with brine, then dried over Na_2_SO_4_. After filtration, the residue was passed through a short silica gel column (eluate: hexane–ethyl acetate = 1:1). After the solvents were removed under reduced pressure, the residue was dissolved in CCl_4_–CH_3_CN–H_2_O (2:2:3, 3 mL). To this solution, were added RuCl_3_·3H_2_O (2.7 mg) and NaIO_4_ (73 mg, 0.34 mmol) at 0 °C. The mixture was stirred at the same temperature for 1.5 h. After diluted with ether, the mixture was washed with water, *sat*.NaHCO_3_ and brine, then dried over Na_2_SO_4_. After filtration, the solvents were removed under reduce pressure, the residue was purified by silica gel column chromatography (hexane-ethyl acetate = 6:1) to give **13** (82 mg, 77%). ^1^H-NMR (CDCl_3_) δ1.43 (3H, s), 1.50 (3H, s), 3.56 (1H, dd, *J* = 12.1, 2.4 Hz), 3.82 (1H, d, *J* = 12.1 Hz), 3.91 (1H, d, *J* = 12.1 Hz), 3.97 (1H, dd, *J* = 12.6, 4.4 Hz), 4.06 (1H, dd, *J* = 12.1, 2.4 Hz), 4.28 (1H, d, *J* = 12.6 Hz), 5.42 (1H, d, *J* = 6.3 Hz), 5.48 (1H, t, *J* = 5.1 Hz); ^13^C-NMR (CDCl_3_) δ 19.6, 27.1, 61.0, 62.4, 69.7, 79.0, 83.3, 84.5, 99.2; IR (KBr) 3000.8, 2892.3, 1699.7, 1380.9, 1089.9 cm^−1^; EI-MS (*m/z*): 267 [M+1]^+^; HRMS Calcd for C_9_H_15_N_3_O_4_: 266.0460, Found: 266.0467.

(3R*,4S*)-3-Azido-8,8-dimethyl-1,7,9,-trioxaspiro[4.5]decan-4-ol (**18**). A mixture of **7** [11,14] (433 mg, 2.33 mmol) and NaN_3_ (752 mg 11.6 mmol) in 2-methoxyethanol (26 mL) was kept at 100 °C for 5 h. After the solvent was removed under reduced pressure, the residue was dissolved in ethyl acetate. After washed with water and brine, the organic layer was dried over Na_2_SO_4_. After filtration, the solvents were removed under reduce pressure, the residue was purified by silica gel column chromatography (hexane–ethyl acetate = 5:1) to give **18** (351 mg, 66%). ^1^H-NMR (CDCl_3_) δ 1.40 (3H, s), 1.49 (3H, s), 3.69-3.80 (3H, m), 3.84 (1H, d, *J* = 11.6 Hz), 4.01–4.08 (3H, m), 4.38 (1H, t, *J* = 1.9, 2.4 Hz); ^13^C-NMR (CDCl_3_) δ 19.5, 27.4, 62.3, 66.0, 67.6, 69.4, 78.2, 79.1, 98.5; IR (neat) 3419.0, 2104.8, 1086.6, 831.7 cm^−1^; EI-MS (*m/z*): 229 [M+1]^+^; HRMS Calcd for C_9_H_15_N_3_O_4_: 229.1063, Found: 229.1062.

(3R*,4S*)-3-Azido-8,8-dimethyl-1,7,9,-trioxaspiro[4.5]decan-4-yl benzoate (**19**). To a solution of **18** (432 mg, 1.88 mmol), Et_3_N (0.59 mL, 4.24 mmol), and DMAP (23 mg, 0.19 mmol) in CH_2_Cl_2_ (15 mL) was added benzoyl chloride (0.40 mL, 3.39 mmol) and the mixture was stirred at room temperature for 6.5 h. The reaction was quenched by addition of MeOH, and the whole was stirred at room temperature for 10 min. The mixture was diluted with CH_2_Cl_2_ and washed with water and brine, then dried over Na_2_SO_4_. After filtration, the solvents were removed under reduce pressure, the residue was purified by silica gel column chromatography (hexane–ethyl acetate = 4:1) to give **19** (598 mg, 95%). ^1^H-NMR (CDCl_3_) δ 1.37 (3H, s), 1.47 (3H, s), 3.84-3.91 (3H, m), 3.99 (1H, dd, *J* = 1.4, 10.6 Hz), 4.07 (1H, dd, *J* = 1.4, 10.6 Hz), 4.18-4.25 (2H, m) 5.47 (1H, d, *J* = 1.0 Hz), 7.48 (2H, t, *J* = 7.2 Hz), 7.62 (1H, *J* = 7.2 Hz), 8.03 (2H, *J* = 7.2 Hz); ^13^C-NMR (CDCl_3_) δ 22.4, 24.2, 62.1, 65.0, 66.2, 69.9, 78.9, 79.2, 98.4, 128.6, 129.0, 129.6, 133.6, 165.3; IR (neat) 2993.1, 2107.2, 1725.4, 1267.4, 1091.3, 711.5 cm^−1^; EI-MS (*m/z*): 333 [M]^+^; HRMS Calcd for C_16_H_19_N_3_O_5_: 333.1325, Found: 333.1336.

(3S*,4R*)-4-Azido-2,2-bis((methylsulfonyloxy)methyl)tetrahydrohuran-3-yl benzoate (**20**). A mixture of **19** (1.01 g, 3.04 mmol) in 80% AcOH (80 mL) was stirred at room temperature for 5 h. After the solvent was removed under reduced pressure, the residue was co-evaporated with EtOH five times to remove residual AcOH. The resulting crude product was dissolved in CH_2_Cl_2_ (40 mL). To this mixture, were added, MsCl (1.19 mL, 15.18 mmol), Et_3_N (2.14 mL, 15.18 mmol), and DMAP (38 mg, 0.30 mmol). After stirred at room temperature for 1 h, the mixture was diluted with CH_2_Cl_2_ washed with 5%HCl, sat.NaHCO_3_ and brine. The separated organic layer was dried over Na_2_SO_4_. After filtration, the solvents were removed under reduce pressure, the residue was purified by silica gel column chromatography (hexane–ethyl acetate = 2:1) to give **20** (1.05 g, 77%). ^1^H-NMR (CDCl_3_) δ 3.00 (3H, s), 3.12 (3H, s), 3.94 (1H, dd, *J* = 5.80, 4.37 Hz), 4.33~4.48 (6H, m), 5.48 (1H, d, *J* = 3.4 Hz), 7.50 (2H, t, *J* = 7.7 Hz), 7.64 (1H, t, *J* = 8.0 Hz), 8.04 (2H, d, *J* = 7.3 Hz); ^13^C-NMR (CDCl_3_) δ 37.7, 65.5, 65.8, 67.4, 70.3, 77.2, 78.9, 82.8, 128.1, 128.8, 129.9, 134.2, 165.2; IR (near) 2110.7, 1728.8, 1360.0, 1267.0 cm^−1^; FAB-MS (*m/z*): 450 [M+1]^+^; HRMS Calcd for C_15_H_20_N_3_O_9_S_2_: 450.0641, Found: 450.0631.

((1R*,4R*,5S*)-4-Azido-2,6-dioxabicyclo[3.2.0]heptan-1-yl)methyl methanesulfonate (**21**). A mixture of **20** (36 mg, 0.08 mmol) and NaOCH_3_ (4.6 mg, 0.08 mmol) in MeOH (2 mL) was kept at 75 °C overnight. After the solvent was removed under reduced pressure, the residue was dissolved in CHCl_3_ and washed with water and brine, then dried over Na_2_SO_4_. After filtration, the solvents were removed under reduce pressure, the residue was purified by silica gel column chromatography (hexane–ethyl acetate = 2:1) to give **21** (14 mg, 72%). ^1^H-NMR (CDCl_3_) δ 3.09 (3H, s), 4.05 (1H, d, *J* = 3.4 Hz), 3.40 (1H, d, *J* = 11.1 Hz), 4.29~4.77 (4H, m), 4.76 (1H, d, *J* = 8.2 Hz), 5.06 (1H, s); ^13^C-NMR (CDCl_3_) δ 37.8, 63.6, 67.4, 71.9, 77.1, 84.4, 89.9; IR (neat) 2098.2, 1360.4, 1175.0, 960.2, 815.5 cm^−1^; FAB-MS (*m/z*): 250 [M+1]^+^; HRMS Calcd for C_7_H_12_N_3_O_5_S: 250.0498, Found: 250.0490.

((1R*,4R*,5S*)-4-Azido-2,6-dioxabicyclo[3.2.0]heptan-1-yl)methyl benzoate (**22**). A mixture of CsF (332 mg, 2.19 mmol) and PhCOOH (267 mg, 2.19 mmol) in DMF (40 mL) was stirred at room temperature for 20 min. To this mixture was added a solution of **21** (182 mg, 0.73 mmol) in DMF (20 mL). After stirred at 60 °C overnight, the mixture was partitioned between EtOAc and H_2_O. The separated water layer was extracted with EtOAc, and the organic layer was washed with *sat.*NaHCO_3_, brine, then dried over Na_2_SO_4_. After filtration, the filtrated was concentrated under reduced pressure. The residue was purified by silica gel column chromatography (hexane-ethyl acetate = 5:1) to give **22** (170 mg, 86%). ^1^H-NMR (CDCl_3_) δ 4.08 (1H, d, *J* = 3.4 Hz), 4.42 (1H, d, *J* = 10.6 Hz), 4.52 (2H, dd, *J* = 10.6, 3.4 Hz), 4.60 (2H, s), 4.83 (1H, d, *J* = 7.7 Hz), 5.15 (1H, s), 7.61 (2H, t, *J* = 15.5 Hz) 7.59 (1H, t, *J* = 17.4 Hz), 8.10 (2H, d, *J* = 9.7 Hz); ^13^C-NMR (CDCl_3_) δ 63.1, 63.8, 71.5, 77.8, 85.1, 90.4, 128.4, 129.4, 129.7, 133.3, 166.1; IR (neat) 2099.7, 1723.1, 1284.3, 713.4 cm^−1^; FAB-MS (*m/z*); 276 [M+1]^+^; HRMS Calcd for C_13_H_14_N_3_O_4_: 276.0984, Found: 276.0977.

((1S*,4R*,5S*)-4-Azido-2,6-dioxa-bicyclo[3.2.0]heptan-1-yl)methanol (**23**). A mixture of **22** (184 mg, 0.67 mmol) and NaOCH_3_ (19 mg, 0.33 mmol) in MeOH (15 mL) was stirred at room temperature. After the mixture was neutralized with AcOH (19 µL), the solvents were removed under reduced pressure and the residue was purified by silica gel column chromatography (hexane–ethyl acetate = 4:1) to give **23** (90 mg, 79%). ^1^H-NMR (CDCl_3_) δ 3.91 (2H, d, *J* = 6.3 Hz), 4.04 (1H, d, *J* = 3.9 Hz), 4.36 (1H, d, *J* = 10.6 Hz), 4.48 (2H, dd, *J* = 10.6, 3.4 Hz), 4.48 (1H, d, *J* = 8.2 Hz), 4.68 (1H, d, *J* = 8.2 Hz), 5.05 (1H, s); ^13^C-NMR (CDCl_3_) δ 62.3, 64.0, 71.5, 77.6, 86.9, 90.2; IR (neat) 3431.5, 2101.6, 1248.2, 870.88 cm^−1^; EI-MS (*m/z*): 171 [M]^+^; HRMS Calcd for C_6_H_9_N_3_O_3_: 171.0644, Found: 171.0643.

((1S*,4R*,5S*)-4-(5-Amino-6-chloropyrimidin-4-ylamino)-2,6-dioxa-bicyclo[3.2.0]heptan-1-yl)methanol (**15**). A mixture of **23** (69 mg, 0.40 mmol) and Pd(OH)_2_ (6.2 mg, 0.04 mmol) in MeOH (5 mL) was stirred at room temperature overnight under H_2_ atmosphere. After insoluble materials were removed by filtration, the solvents were removed under reduced pressure. The resulting crude product was dissolved in *n*-BuOH (3 mL). To this mixture, were added 5-amino-4,6-dichloropyrimidine (140.1 mg, 0.86 mmol) and *i*-Pr_2_NEt (298 µL, 1.71 mmol). The mixture was kept under reflux overnight. After the solvents were removed under reduced pressure, the residue was purified by silica gel column chromatography (chloroform–methanol = 19:1) to give **15** (64 mg, 58%). ^1^H-NMR (CD_3_OD) δ3.67 (2H, q, *J* = 12.6 Hz), 4.24 (1H, d, *J* = 10.1 Hz), 4.38 (1H, d, *J* = 7.7 Hz), 4.45 (1H, d, *J* = 4.4 Hz), 4.50 (1H, d, *J* = 10.1, 4.4 Hz), 4.63 (1H, d, *J* = 7.2 Hz) 4.90 (1H, s), 7.72 (1H, s); ^13^C NMR (CD_3_OD) δ 58.1, 58.3, 62.7, 73.1, 88.3, 92.1, 125.5, 138.9, 147.3, 153.3; IR (KBr) 3381.3, 2926.7, 1578.6, 1056.3 cm^−1^; EI-MS (*m/z*): 272 [M]^+^; HRMS Calcd for C_10_H_13_ClN_4_O_3_: 272.0676, Found: 272.0673.

((1S*,4R*,5S*)-4-(6-Chloro-9H-purin-9-yl)-2,6-dioxa-bicyclo[3.2.0]heptan-1-yl)methanol (**24**). To a solution of **15** (18 mg, 0.07 mmol) in DMF (0.5mL), were added orthoethyl formate (0.7 mL, 4.21 mmol) and *conc* HCl (2 µL, 0.024 mmol) at 0 °C. After the mixture was stirred at room temperature, the solvents were removed under reduced pressure. The residue was dissolved in 0.5 M *aq*HCl (1 mL) and the mixture was stirred at room temperature for 1 h. The mixture was neutralized with 1M aqNaOH (0.5 mL) and concentrated under reduced pressure. The residue was extracted with a solution of chloroform–methanol = 1:1. After the insoluble materials were removed by filtration, the solvents were removed under reduced pressure. The residue was purified by *p*TLC (developed by chloroform-methanol = 5:1) to give **24** (14.9 mg, 81%). ^1^H-NMR (CDCl_3_) δ 4.12 (2H, q, *J* = 12.6 Hz), 4.59 (1H, d, *J* = 11.6 Hz), 4.66 (1H, d, *J* = 9.7 Hz), 4.68 (1H, d, *J* = 9.7 Hz), 4.66 (1H, d, *J* = 9.7 Hz), 4.90 (1H, dd, *J* = 11.1, 4.8 Hz), 5.24 (1H, s), 5.38 (1H, d, *J* = 4.4 Hz), 8.68 (1H, s), 8.76 (1H, s); ^13^C-NMR (CDCl_3_-CD_3_OD = 19 : 1) δ 58.6, 61.2, 71.9, 77.2, 87.8, 90.9, 130.7, 144.6, 150.7, 151.2, 151.8; IR (KBr) 3401.4, 2931.1, 1597.4, 1567.4, 1056.6 cm^−1^; EI-MS (*m/z*): 282 [M]^+^; HRMS Calcd for C_11_H_11_ClN_4_O_3_: 282.0520, Found: 282.0506.

((1S*,4R*,5S*)-4-(6-Amino-9H-purin-9-yl)-2,6-dioxa-bicyclo[3.2.0]heptan-1-yl)methanol (**6**). Compound **24** (29.4 mg, 0.10 mmol) was dissolved in *sat*. methanolic ammonia (7 mL) and the mixture was kept at 100 °C for 21 h in a glass sealed tube. After the solvents were removed under reduced pressure, the residue was purified by *p*TLC (developed by chloroform–methanol = 5:1) to give **6** (18.8 mg, 69%). ^1^H-NMR (CDCl_3_-CD_3_OD = 17:3) δ 3.91 (1H, d, *J* = 12.6 Hz), 4.00 (1H, d, *J* = 12.1 Hz), 4.57 (1H, d, *J* = 10.6 Hz), 4.64 (1H, d, *J* = 7.7 Hz), 4.70 (1H, d, *J* = 7.7 Hz), 4.87 (1H, dd, *J* = 11.1, 4.8 Hz), 5.19 (1H, s), 5.25 (1H, d, *J* = 4.8 Hz), 8.25 (1H, s), 8.28 (1H, s); ^13^C-NMR (CDCl_3_–CD_3_OD = 17:3) δ 29.5, 58.3, 61.3, 72.1, 87.6, 91.0, 118.2, 139.1, 148.9, 152.6, 155.3; IR (KBr) 3192.2, 2409.9, 1660.5, 1615.0, 1054.9 cm^−1^; EI-MS (*m/z*): 263 [M]^+^; HRMS Calcd for C_11_H_1__3_N_5_O_3_: 263.1018, Found: 263.1021.

## 4. Conclusions

We constructed an isoadenosine derivative on a 2,6-dioxobicyclo[3.2.0]heptane scaffold. Since our initial attempt to synthesize **6** by directly introducing the adenine moiety was not successful, we synthesized it by the de novo synthesis of an adenine ring on a pseudosugar moiety. However, this unique adenosine analogue showed no activity against HIV. Previously, we have reported that neither thymine nor adenine analogues **4** built on a 2-oxa-6-thiabicyclo[3.2.0]heptane skeleton inhibit HIV [14]. The structural rigidities of these analogues and isoadenosine **6** due to the introduction of fused thietane and oxetane rings, respectively, appear to inhibit anti-HIV activity. In particular, phosphorylation at the 5'-hydroxyl group would be inhibited since deoxynucleoside kinase recognizes the puckering of sugars [25]. Thus, we are currently preparing new substituted nucleoside derivatives based on **4** and **6**, and the results will be reported elsewhere.

## References

[1] Cihlar T., Ray A.S. (2010). Nucleoside and nucleotide HIV reverse transcriptase inhibitors: 25 years after zidovudine. Antivir. Res..

[2] Mehellou Y., de Clercq E. (2010). Twenty-six years of anti-HIV drug discovery: Where do we stand and where do we go?. J. Med. Chem..

[3] WHO Consolidated Guidelines on the Use of Antiretroviral Drugs for Treating and Preventing HIV Infection. http://www.who.int/hiv/pub/guidelines/arv2013/download/en/.

[4] Meadows D.C., Gervey-Hague J. (2006). Current developments in HIV chemotherapy. ChemMedChem.

[5] Imamichi T. (2004). Action of anti-HIV drugs and resistance: Reverse transcriptase inhibitors and protease inhibitors. Curr. Pharm. Des..

[6] Mitsuya H., Broder S. (1986). Inhibition of the *in vitro* infectivity and cytopathic effect of human T-lymphotrophic virus type III/lymphadenopathy-associated virus (HTLV-III/LAV) by 2',3'-dideoxynucleosides. Proc. Natl. Acad. Sci. USA.

[7] Mitsuya H., Weinhold K.J., Furman P.A., St Clair M.H., Lehrman S.N., Gallo R.C., Bolognesi D., Barry D.W., Broder S. (1985). 3'-Azido-3'-deoxythymidine (BW A509U): An antiviral agent that inhibits the infectivity and cytopathic effect of human T-lymphotropic virus type III/lymphadenopathy-associated virus *in vitro*. Proc. Natl. Acad. Sci. USA.

[8] Schinazi R.F., Chu C.K., Peck A., McMillan A., Mathis R., Cannon D., Jeong L.S., Beach J.W., Choi W.B., Yeola S. (1992). Activities of the four optical isomers of 2',3'-dideoxy-3'-thiacytidine (BCH-189) against human immunodeficiency virus type 1 in human lymphocytes. Antimicrob. Agents Chemother..

[9] Balzarini J., Holy A., Jindrich J., Naesens L., Snoeck R., Schols D., de Clercq E. (1993). Differential antiherpesvirus and antiretrovirus effects of the (*S*) and (*R*) enantiomers of acyclic nucleoside phosphonates: Potent and selective *in vitro* and *in vivo* antiretrovirus activities of (*R*)-9-(2-phosphonomethoxypropyl)-2,6-diaminopurine. Antimicrob. Agents Chemother..

[10] Yoshimura Y., Yamazaki Y., Kawahata M., Yamaguchi K., Takahata H. (2007). Design and synthesis of a novel ring-expanded 4'-Thio-*apio*-nucleoside derivatives. Tetrahedron Lett..

[11] Yoshimura Y., Asami K., Matsui H., Tanaka H., Takahata H. (2006). New synthesis of (±)-isonucleosides. Org. Lett..

[12] Yoshimura Y., Yamazaki Y., Saito Y., Takahata H. (2009). Synthesis of 1-(5,6-dihydro-2H-thiopyran-2-yl)uracil by a Pummerer-type thioglycosylation reaction: The regioselectivity of allylic substitution. Tetrahedron.

[13] Yoshimura Y., Ohta M., Imahori T., Imamichi T., Takahata H. (2008). A new entry to carbocyclic nucleosides: Oxidative coupling reaction of cycloalkenylsilanes with a nucleobase mediated by hypervalent iodine reagent. Org. Lett..

[14] Yoshimura Y., Asami K., Imamichi T., Okuda T., Shiraki K., Takahata H. (2010). Design and synthesis of isonucleosides constructed on a 2-oxa-6-thiabicyclo[3.2.0]heptane scaffold. J. Org. Chem..

[15] Yoshimura Y., Yamazaki Y., Saito Y., Natori Y., Imamichi T., Takahata H. (2011). Synthesis of 5-thiodidehydropyranylcytosine derivatives as potential anti-HIV agents. Bioorg. Med. Chem. Lett..

[16] Kiran Y.B., Wakamatsu H., Natori Y., Takahata H., Yoshimura Y. (2013). Design and synthesis of a nucleoside and a phosphonate analogue constructed on a branched-*threo*-tetrofuranose skeleton. Tetrahedron Lett..

[17] Kan-no H., Saito Y., Omoto S., Minato S., Wakamatsu H., Natori Y., Imamichi T., Takahata H., Yoshimura Y. (2014). Synthesis of a dihydropyranonucleoside using an oxidative glycosylation reaction mediated by hypervalent iodine. Synthesis.

[18] Chu C.K., Ahn S.K., Kim H.O., Beach J.W., Alves A.J., Jeong L.S., Islam Q., van Roey P., Schinazi R.F. (1991). Asymmetric synthesis of enantiomerically pure (−)-(1'R,4'R)-dioxolane-thymine and its anti-HIV activity. Tetrahedron Lett..

[19] Kim H.O., Schinazi R.F., Nampalli S., Shanmuganathan K., Cannon D.L., Alves A.J., Jeong L.S., Beach J.W., Chu C.K. (1993). 1,3-dioxolanylpurine nucleosides (2*R*,4*R*) and (2*R*,4*S*) with selective anti-HIV-1 activity in human lymphocytes. J. Med. Chem..

[20] Kim H.O., Schinazi R.F., Shanmuganathan K., Jeong L.S., Beach J.W., Nampalli S., Cannon D.L., Chu C.K. (1993). l-beta-(2*S*,4*S*)- and l-alpha-(2*S*,4*R*)-dioxolanyl nucleosides as potential anti-HIV agents: Asymmetric synthesis and structure-activity relationships. J. Med. Chem..

[21] Choi Y., George C., Comin M.J., Brachi J.J., Kim H.S., Jacobsen K.A., Balzarini J., Mitsuya H., Boyer P.L., Hughes S.H. (2003). A conformationally locked analogue of the anti-HIV agent stavudine. An important correlation between pseudorotation and maximum amplitude. J. Med. Chem..

[22] D’Alonzo D., van Aerschot A., Guaragna A., Palumbo G., Schepers G., Capone S., Rozenski J., Herdewijn P. (2009). Synthesis and base pairing properties of 1',5'-anhydro-l-hexitol nucleic acids (l-HNA). Chem. Eur. J..

[23] Bera S., Nair V. (2001). A new general synthesis of isomeric nucleosides. Tetrahedron Lett..

[24] Quadrelli P., Piccanello A., Mella M., Corsaro A., Pistarà V. (2008). From cyclopentadiene to isoxazoline-carbocyclic nucleosides: A rapid access to biological molecules through aza-Dielse Alder reactions. Tetrahedron.

[25] Comin M.J., Vu B.C., Boyer P.L., Liao C., Hughes S.H., Marquez V.E. (2008). d-(+)-iso-methanocarbathymidine: A high-affinity substrate for herpes simplex virus 1 thymidine kinase. ChemMedChem.

